# Piscidin-1-analogs with double L- and D-lysine residues exhibited different conformations in lipopolysaccharide but comparable anti-endotoxin activities

**DOI:** 10.1038/srep39925

**Published:** 2017-01-04

**Authors:** Amit Kumar, Mukesh Mahajan, Bhanupriya Awasthi, Anshika Tandon, Munesh Kumar Harioudh, Sonal Shree, Pratiksha Singh, Praveen Kumar Shukla, Ravishankar Ramachandran, Kalyan Mitra, Surajit Bhattacharjya, Jimut Kanti Ghosh

**Affiliations:** 1 Molecular and Structural Biology Division, CSIR-Central Drug Research Institute, Sector 10, Jankipuram Extension, Sitapur Road, Lucknow–226 031, India; 2 School of Biological Sciences, Nanyang Technological University, 60 Nanyang Drive, Singapore 637551, Singapore; 3 Electron Microscopy Unit, CSIR-Central Drug Research Institute, Sector 10, Jankipuram Extension, Sitapur Road, Lucknow–226 031, India; 4 Microbiology Division, CSIR-Central Drug Research Institute, Sector 10, Jankipuram Extension, Sitapur Road, Lucknow–226 031, India

## Abstract

To become clinically effective, antimicrobial peptides (AMPs) should be non-cytotoxic to host cells. Piscidins are a group of fish-derived AMPs with potent antimicrobial and antiendotoxin activities but limited by extreme cytotoxicity. We conjectured that introduction of cationic residue(s) at the interface of polar and non-polar faces of piscidins may control their insertion into hydrophobic mammalian cell membrane and thereby reducing cytotoxicity. We have designed several novel analogs of piscidin-1 by substituting threonine residue(s) with L and D-lysine residue(s). L/D-lysine-substituted analogs showed significantly reduced cytotoxicity but exhibited either higher or comparable antibacterial activity akin to piscidin-1. Piscidin-1-analogs demonstrated higher efficacy than piscidin-1 in inhibiting lipopolysaccharide (LPS)-induced pro-inflammatory responses in THP-1 cells. T15,21K-piscidin-1 (0.5 mg/Kg) and T15,21dK-piscidin-1 (1.0 mg/Kg) demonstrated 100% survival of LPS (12.0 mg/Kg)-administered mice. High resolution NMR studies revealed that both piscidin-1 and T15,21K-piscidin-1 adopted helical structures, with latter showing a shorter helix, higher amphipathicity and cationic residues placed at optimal distances to form ionic/hydrogen bond with lipid A of LPS. Remarkably, T15,21dK-piscidin-1 showed a helix-loop-helix structure in LPS and its interactions with LPS could be sustained by the distance of separation of side chains of R7 and D-Lys-15 which is close to the inter-phosphate distance of lipid A.

Antimicrobial peptides (AMPs) are not only known for their antimicrobial activity but also are now widely documented for their multifunctional roles in both innate and adaptive immune responses and have been identified in all classes of life^1^,^2^. These peptides show potent broad spectrum activity against microorganisms that include Gram-negative and Gram-positive bacteria, enveloped viruses, fungi and even cancerous cells and display potential for their future development as novel therapeutic agents^3^,^4^,^5^,^6^,^7^,^8^,^9^,^10^.

Piscidins belong to a novel family of broad spectrum AMPs that were identified in hybrid striped bass, an important aquaculture fish. NMR studies suggest that piscidins adopt an amphipathic α–helical conformation upon interaction with model membranes^11^ or detergent micelles^5^,^12^. Piscidin-1 possesses LPS-neutralizing property^13^,^14^, and has potent activity against a variety of microbes, comprising of filamentous fungi, yeast, and Gram-positive and negative bacteria and their resistant versions. However, this peptide causes haemolysis of RBCs and is cytotoxic, which may limit its therapeutic applications^15^,^16^,^17^,^18^,^19^. Therefore, it was of our interest to design novel analogs of piscidin-1 with antibiotic/anti-inflammatory properties but reduced cytotoxicity to normal mammalian cells.

Introduction of cationic residues within an AMP could strongly influence its biological properties. Though there are numerous reports^20^,^21^,^22^ on introduction of cationic residues in the polar faces of AMPs, impact of introduction of cationic residues precisely in the interface of non-polar and polar faces is not well known. Further, we envisioned that placement of cationic residues just outside its non-polar faces or at the interfaces of hydrophobic and hydrophilic faces of piscidin-1 could weaken the interaction between its hydrophobic face and hydrophobic outer membrane of mammalian cell, resulting in its weaker penetration in the mammalian cell membrane and thus reducing its cytotoxicity. Interestingly, we found that the amino acids located at the right as well as left interface of hydrophobic and hydrophilic faces or immediately outside the hydrophobic faces of piscidin-1 are two threonine residues (at 15^th^ and 21^st^ positions). Therefore, we have rationalized that Thr to Lys substitutions at both the interfaces of piscidin-1 might yield membrane selective AMP analogs killing only microorganisms.

The impact of substitution of single threonine residue by a lysine residue in the interface of non-polar and polar faces was also investigated. Since many times incorporation of D-amino acids in AMPs results in the reduction of their cytotoxic properties^23^,^24^, by perturbing their helical structures, the effect of substitution with D-lysine residue(s) instead of L-lysine residue(s) in the interface of polar and non-polar faces of piscidin-1 was also investigated. Results show that substitutions of lysine residues in piscidin-1 significantly impair its cytotoxicity without affecting its antibacterial and anti-endotoxin properties. High resolution NMR structures were determined for piscidin-1, its double L-lysine and D-lysine substituted analogs in LPS to understand their interactions with LPS, the basis of their anti-endotoxin properties as well as the influence of double L and D- lysine substitutions in the atomic structure of piscidin-1 in LPS.

## Results

### Design of piscidin-1 analogs

Piscidin-1 adopts an α-helical structure from Phe^2^ to Thr^21^ in SDS micelles as detected by NMR spectroscopy^5^. The helical wheel diagram of piscidin-1, drawn by ‘Protein ORIGAMI’ tool, and shown in Supplementary Figure S1, indicates its significant amphipathic character due to presence of a long hydrophobic face as well as hydrophilic face with appreciable number of polar residues. However, the hydrophobic sector of piscidin-1 appears significantly longer than its hydrophilic sector which may contribute to its high cytotoxicity^25^. Moreover, piscidin-1 contains only three cationic residues at physiological pH which is relatively small considering that it contains 22-residues. Thus several analogs of piscidin-1 were designed after incorporating additional L or D cationic lysine residue(s) in it (Table 1) as described in the last section of ‘Introduction’ and synthesized along with the native peptide. Piscidin-1-analogs containing D-amino acids have been omitted in the helical wheel diagrams since these amino acids are known to impair the helical structure of a peptide^23^,^24^. Observed molecular mass of these peptides, determined by MALDI-TOF experiments, were close to that of the calculated mass of the respective peptides confirming the syntheses of the correct peptides (Table 1). HPLC retention times for purification of piscidin-1 and its analogs are shown in Table 1 and their HPLC profiles are shown in Supplementary Figure S2.

The physicochemical parameters of piscidin-1 and its analogs are shown in Supplementary Table S1. The substitution of threonine residue(s) with cationic lysine residue(s) results in the decrease of hydrophobic character of piscidin-1 as evidenced by the hydrophobicity and GRAVY values (Supplementary Table S1). Interestingly, introduction of lysine residues to some extent increased the amphipathic properties of piscidin-1 as indicated by the hydrophobic moment values. Overall there were decrease in hydrophobicity and increase in amphipathicity of piscidin-1 following the substitution of threonine residue(s) with lysine residue(s) in its interface of hydrophobic and hydrophilic faces (Supplementary Table S1).

### Substitution of threonine residues at 15^th^ and/or 21^st^ position with L- or D-lysine residue(s) significantly reduced the cytotoxicity of piscidin-1

**Figure 1:**
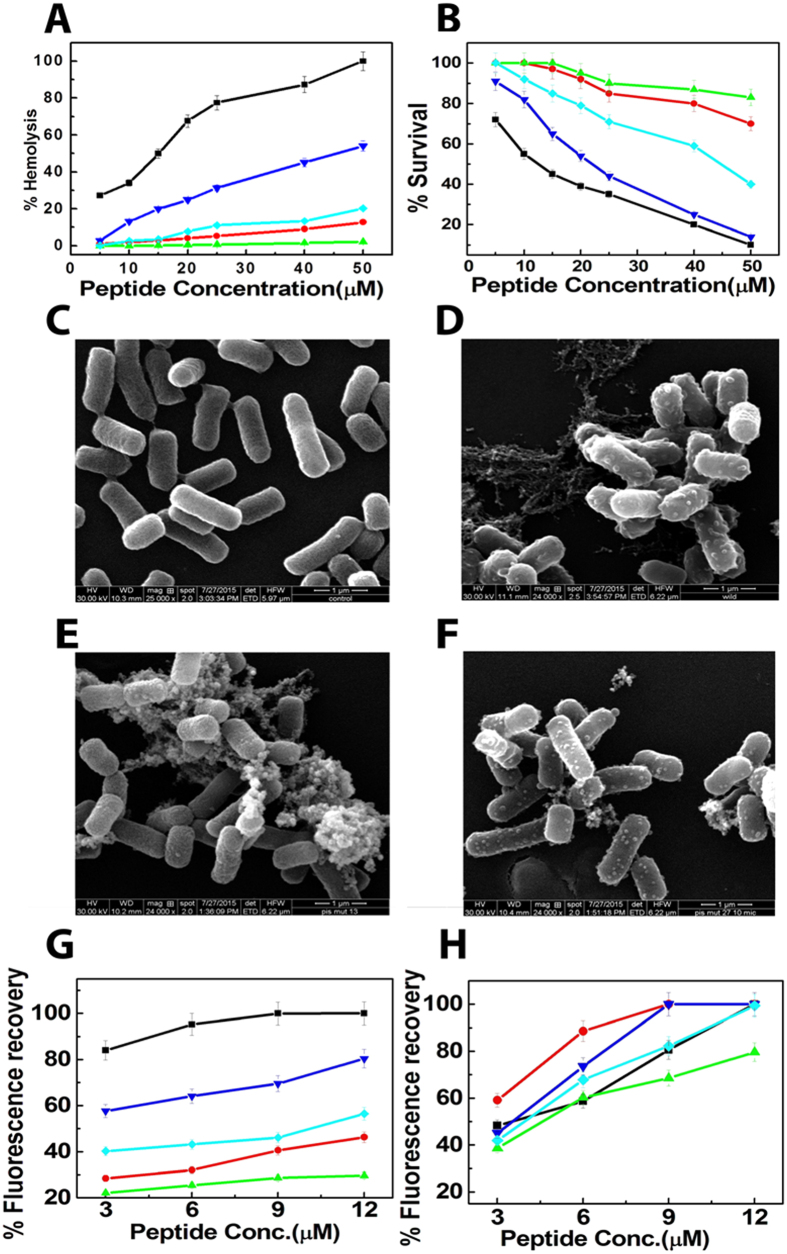
(**A**) and (**B**) Show the dose-dependent hemolysis of hRBCs and viability of murine 3T3 cells respectively in the presence of piscidin-1 and its analogs. (**C** to **F**) Scanning Electron Microscopy of *E. coli* ATCC 25922 in the absence and presence of piscidin-1 and its selected analogs; bacteria without any treatment (**C**), Bacteria after treatment with piscidin-1, T15,21 K piscidin-1 and T15,21dK piscidin-1 at 10 fold MIC of the respective peptide (**D**,**E** and **F**). (**G**) and (**H**) Show the plot of fluorescence recovery which is a measure of peptide-induced membrane permeabilization vs peptide concentration (μM) in mammalian and bacterial membrane mimetic PC/Chol (8:1, w/w) and PC/PG (3:1, w/w) lipid vesicles respectively. **Symbols**: Black square, piscidin-1; Red circle, T15,21K-piscidin-1; Green up triangle, T15,21dK-piscidin-1; Blue down triangle, T15K-piscidin-1 and Cyan diamond, T15dK-piscidin-1. Each data point is an average of three independent experiments and error bar represents the standard deviation.

Cytotoxic activity of piscidin-1 and its designed analogs was examined by assaying the lysis of human red blood cells (hRBCs) and the viability of murine 3T3 cells in presence of these peptides. Piscidin-1 showed a complete lyses (~100%) of hRBCs at 50 μM as was reported in earlier studies^5^,^15^ while its double lysine-substituted analog, T15,21K-piscidin-1 demonstrated significantly reduced haemolytic activity at the same concentration. Introduction of D-lysine (dK)residues instead of threonine residues at 15^th^ & 21^st^ positions further reduced the haemolytic activity of piscidin-1 (Fig. 1A). Overall, haemolytic activity of these peptides against hRBCs followed the order piscidin-1 > T15K-piscidin-1 > T15dK-piscidin-1 > T15,21K-piscidin-1 > T15,21dK-piscidin-1. Viability of 3T3 cells as determined by MTT assay in the presence of these peptides followed the same trend as their haemolytic activity (Fig. 1B). The results altogether indicated that substitution of threonine residue(s) in the interface of hydrophobic and hydrophilic faces of piscidin-1 with L-/D-lysine residue(s) significantly reduced its cytotoxicity.

### The designed analogs substantially retained the antibacterial property of native piscidin-1

Piscidin-1 and its analogs were examined for bacterial growth-inhibiting activity in liquid cultures against Gram-positive bacteria including MRSA strains and Gram-negative bacteria (Table 2). Piscidin-1 showed significant antibacterial activities. However, the double lysine-substituted, T15,21K-piscidin-1 showed to some extent higher antimicrobial activity than piscidin-1 against majority of the bacterial strains employed here. Particularly, this analog exhibited two fold higher antibacterial activities than piscidin-1 against three of the four MRSA strains used. While T15,21dK-piscidin-1 exhibited 1–2 fold lower activity than the native peptide against most of these strains (Table 2). Thus the results suggested that piscidin-1 substantially retained its antibacterial activity after introduction of cationic L/D-lysine residue(s) at the interface of its polar and non-polar faces. The therapeutic index is a parameter that signify the specificity or selectivity of antimicrobial agents towards microorganisms over mammalian cells^26^. As shown in Supplementary Table S2, T15,21K-piscidin-1 and T15,21dK-piscidin-1 both showed ~20 fold higher therapeutic indices than piscidin-1.

### Visualization of bacterial morphology after the treatments of piscidin-1 and its analogs under the scanning electron microscope

To obtain deeper insight about the mode of action of piscidin-1 and its analogs, morphology of *E. coli* ATCC25922 was visualized by scanning electron microscopy (SEM) after treatment with these peptides. T15,21K-piscidin-1 and T15,21dK-piscidin-1 were employed as representative piscidin-1-analogs. Bacteria, not treated with any peptide appeared as smooth surface under SEM (Fig. 1C). However, *E. coli,* treated with piscidin-1 at 10 fold MIC for 60 min, showed prominent changes in their cellular morphology including wrinkling, surface roughening and membrane blebbing (Fig. 1D). T15,21K-piscidin-1 showed even more drastic changes in the cellular morphology of *E. coli* including extensive damage of membrane, leakage of cellular contents and cell lyses (Fig. 1E). T15,21dK-piscidin-1 also caused appreciable changes in bacterial morphology (Fig. 1F). Altogether the results suggested that piscidin-1 and its double L/D-lysine substituted analogs inflicted considerable damage to *E. coli* membrane and this membrane disruption property could be the basis of antibacterial activity of these peptides.

### Differences between piscidin-1 and its analogs in peptide-induced permeabilization of zwitterionic but not negatively charged lipid vesicles

To comprehend the basis of cytotoxic and antibacterial activities of piscidin-1 and its analogs, peptide-induced permeabilization of mammalian membrane mimetic^27^,^28^ PC/Chol (8:1 w/w) and bacterial membrane mimetic^11^,^14^,^29^, PC/PG (3:1 w/w) lipid vesicles was studied. Matching with its haemolytic/cytotoxic properties, piscidin-1 induced the maximum permeabilization (expressed as the percentage of fluorescence recovery) (Fig. 1G) in PC/Chol lipid vesicles. Piscidin-1-analogs followed the same trend in permeabilizing PC/Chol lipid vesicles as their haemolytic/cytotoxic activity (Fig. 1G). T15,21dK-piscidin-1 showed the least membrane permeabilization in zwitterionic lipid vesicles which is probably indicative of its negligible cytotoxicity. Whereas T15,21K-piscidin-1 permeabilized bacterial membrane mimetic, PC/PG lipid vesicles the maximum supporting its highest antibacterial activity among these peptides (Fig. 1H). Nevertheless, consistent with their anti-bacterial properties, piscidin-1 and its analogs induced appreciable permeabilization in PC/PG lipid vesicles (Fig. 1H).

### Secondary structures of piscidin-1 and its analogs in negatively charged and zwitterionic lipid vesicles

**Figure 2:**
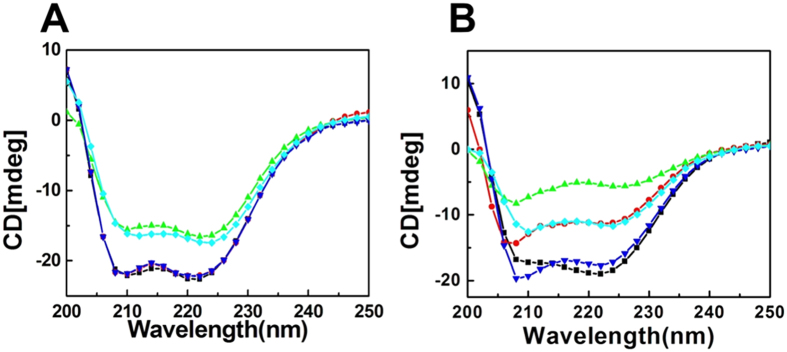
Determination of secondary structures of the piscidin-1 and its analogs, in the presence of (**A**) negatively charged PC/PG lipid vesicles, ~500 μM and (**B**) zwitterionic PC/Chol lipid vesicles, ~500 μM. Concentration of the peptides were ~25 μM. Symbols: Black square, piscidin-1; Red circle, T15,21K-piscidin-1; Green up triangle, T15,21dK-piscidin-1; Blue down triangle, T15K-piscidin-1 and Cyan diamond, T15dK-piscidin-1.

Secondary structures of piscidin-1 and its analogs were assessed in PBS (pH 7.4), negatively charged (PC/PG, 3:1 w/w) and zwitterionic (PC/Chol, 8:1 w/w) lipid vesicles by circular dichroism studies. Since none of these peptides showed any appreciable secondary structures in PBS, the profiles are not shown. However, in the presence of PC/PG lipid vesicles piscidin-1 and its analogs adopted significant helical structures as indicated by the mean residue ellipticity values at 222 nm though the extent of their structures varied to some extent (Fig. 2A; Suppl. Table-S3). Piscidin-1 and the analogs also adopted helical structure in PC/Chol lipid vesicles (Fig. 2B). However, piscidin-1 and its single lysine substituted analog, T15K-piscidin-1, adopted the maximum helical structures in zwitterionic lipid vesicles whereas T15,21K-piscidin-1, T15,21dK-piscidin-1 and T15dK-piscidin-1 exhibited much lower helical structures in the same environment (Suppl. Table-S3). Interestingly, though T15,21K-piscidin-1 showed the highest helical structure in negatively charged lipid vesicles, it showed much lower helix content in the zwitterionic lipid vesicles (Fig. 2B; Suppl. Table-S3) suggesting a significant influence of composition of lipid vesicles on the secondary structure of the peptide.

### Localization of piscidin-1 and its analogs onto bacteria and hRBCs by confocal microscopy

**Figure 3:**
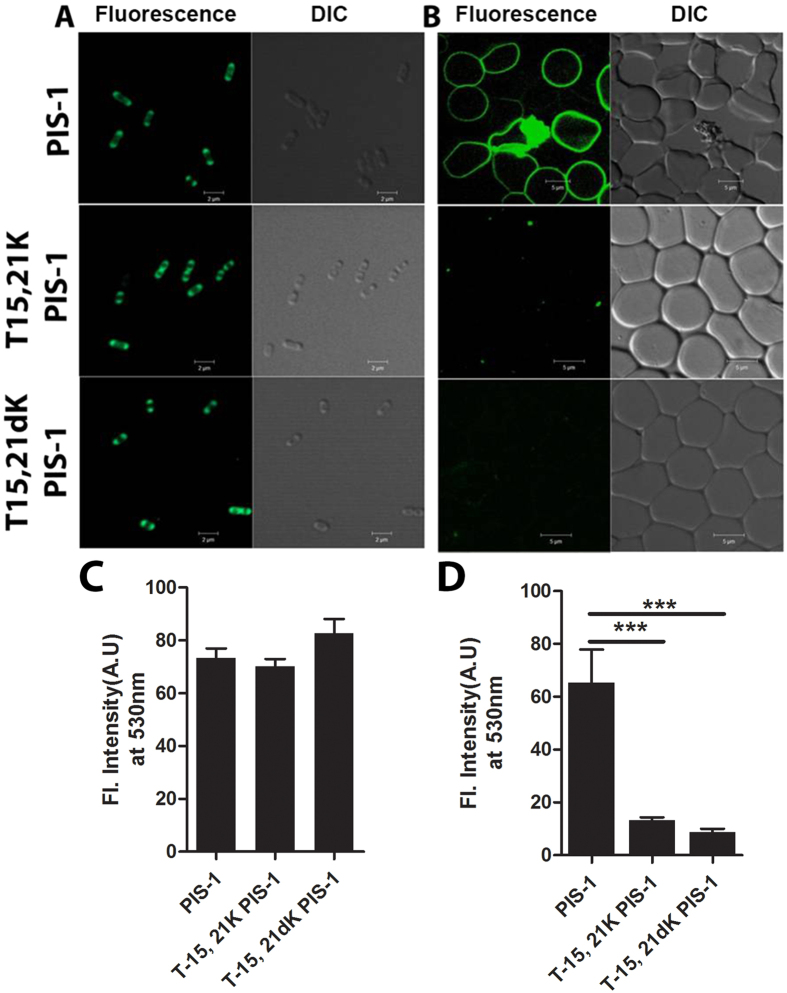
Confocal laser scanning microscopy to look into the localization of piscidin-1 and its selected analogs onto *E. coli* and hRBCs. (**A**) Study of localization of NBD-labelled piscidin-1 and its analogs namely, T15,21K-piscidin-1 and T15,21dK-piscidin-1 (both at ~5.0 μM) onto *E. coli ATCC 25922* and (**B**) study of localization of NBD-labeled piscidin-1 and T15,21K-piscidin-1 and T15,21dK-piscidin-1 (~10.0 μM) onto hRBCs. Fluorescence and DIC images for treatment of different cells with each of the peptides are shown as marked on top of the respective panel. Panels (C) and (D) show the quantitative analysis of fluorescence intensity of *E. coli* and hRBCsbound to NBD-labeled piscidin-1, T15,21K-piscidin-1 and T15,21dK-piscidin-1 respectively.

The cellular localization of piscidin-1 and its analogs onto *E.coli* ATCC 25922 was probed by confocal scanning laser microscopy by employing NBD-labelled versions of piscidin-1^14^ and its two least cytotoxic analogs, T15,21K-piscidin-1 and T15,21dK-piscidin-1. Confocal microscopic images of bacteria following the treatment of NBD-labeled piscidin-1 and its analogs showed appreciable green fluorescence (Fig. 3A) which indicated considerable binding of these peptides onto the bacteria. The data could be indicative of a similar mode of action of piscidin-1 and its non-toxic analogs, T15,21K-piscidin-1 and T15,21dK-piscidin-1 against *E. coli*.

The localization of NBD-labeled piscidin-1, T15,21K-piscidin-1 and T15,21dK-piscidin-1 was also studied onto hRBCs by confocal microscopy^14^. Only NBD-labeled piscidin-1 localized effectively onto the hRBCs, as seen by the prominent green fluorescence on these cell membranes (Fig. 3B). Quantitative analyses of the confocal microscopic fluorescence images were carried out. At least 25 cells from fluorescence images for each set of experiments were included in the analysis. The results are shown at the bottom of the fluorescence images of the respective cells (Fig. 3C and D). Quantitative analysis of fluorescence intensity of *E. coli* bound to different NBD-labeled peptides indicated that NBD-piscidin-1 and its two selected analogs namely NBD-T15,21K-piscidin-1 and NBD-T15,21dK-piscidin-1 possessed comparable binding onto the bacteria. However, the analogs showed significantly lower (p < 0.0001) affinity for hRBCs. Human RBCs bound to NBD-T15,21K-piscidin-1 and NBD-T15,21dK-piscidin-1 showed about 5- fold and 7.5-fold lower fluorescence respectively than the cells bound to NBD-labeled piscidin-1. These results are supportive of antibacterial and haemolytic activities of these peptides.

### LPS-neutralizing activity of the piscidin-1 and its analogs

**Figure 4:**
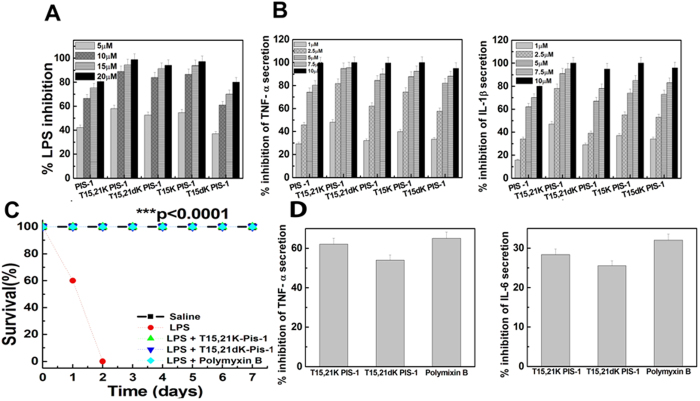
(**A**), Dose dependent LPS neutralization by the piscidin-1 and its analogs as determined by LAL assay; (**B**) the percentage inhibition of levels of LPS-induced secretions of TNF-α and IL-1β, respectively, in the presence of piscidin-1 and its analogs by ELISA experiments; (**C**) *In vivo* anti-LPS activity of the peptides in mice. Septic shock in BALB/c mice was induced by intraperitoneal (i.p.) injection of *E. coli* LPS (12 mg/kg) followed by i.p. injection of T15,21K-piscidin-1 (0.5 mg/Kg) or T15,21dK-piscidin-1 (1 mg/Kg) or polymyxin B (1 mg/Kg) ~5 min later. Mice treated with only saline was taken as control. Survival of the animals (n = 5) was monitored for 7 days (***p < 0.0001, log-rank test). (**D**) Percentage inhibition of TNF-α and IL-6 in LPS (12 mg/Kg) treated mice serum in the presence of piscidin-1 analogs T15,21K-piscidin-1 (0.5 mg/Kg), T15,21dK-piscidin-1 (1 mg/Kg) and polymyxin B (1 mg/Kg) by ELISA experiments.

Dose-dependent ability of piscidin-1-derived peptides to bind or neutralize LPS was determined by chromogenic LAL assay (Fig. 4A). Piscidin-1 showed significant binding to LPS as evidenced by the substantial inhibition of LPS-induced activation of LAL enzyme in its presence. However, piscidin-1-analogs exhibited either higher or similar binding to LPS as compared to the parent peptide. Taken together the binding of piscidin-1 to LPS either increased or retained as a result of introduction of L or D-lysine residue(s) in place of threonine residue(s).

### Piscidin-1-analogs showed comparable or higher *in vitro* LPS neutralization than the parent peptide

Enzyme-linked immunosorbent assays were performed to estimate the secreted TNF-α and IL-1β in LPS-treated THP-1 cells in presence of piscidin-1 and its analogs after 4–6 hr of incubation. Levels of these cytokines in culture supernatant of untreated and LPS-treated cells were taken as minimum and maximum for determining the percentage of inhibition by the peptides. We observed that the levels of these cytokines in cell culture supernatant media in the presence of piscidin-1-analogs were to some extent lower as compared to that in the presence of piscidin-1 at all tested peptide concentrations (Fig. 4B). The results suggested that introduction of L or D-lysine residue(s) in the interface of hydrophobic and hydrophilic faces of piscidin-1 moderately augmented its efficacy to neutralize LPS-induced pro-inflammatory responses in THP-1 cells.

To further investigate the suppression of pro inflammatory cytokine secretion by piscidin-1 and its analogs in THP-1 cells, the concentrations of secreted cytokines (TNF-α, IL-1β, IL-6 and IL-8) in tissue culture supernatant were estimated by cytometric bead array (Supplementary Figure S3). As observed in the previous section, the analogs showed better efficacy in attenuating the production of pro-inflammatory cytokines in LPS-stimulated THP-1 cells than their parent peptide (Supplementary Figure S3).

### Piscidin-1-analogs, T15,21K-piscidin-1 and T15,21dK-piscidin-1 showed comparable efficacy in survival of LPS-administered mice

Piscidin-1-analogs with both high *in vitro* anti-endotoxin activity and low cytotoxicity namely, T15,21K-piscidin-1 and T15,21dK-piscidin-1 were chosen for investigating their *in vivo* anti-endotoxin activities in mice. Polymyxin B was taken as the positive control and the experiments were performed as reported earlier^14^,^30^,^31^. We observed that experimental groups of LPS-treated (12 mg/kg) mice, further administered with single dose of T15,21K-piscidin-1 (0.5 mg/kg) or T15,21dK-piscidin-1 (1.0 mg/kg) or polymyxin B (1.0 mg/kg) resisted the lethal effects of LPS-toxicity with respect to mice groups treated with LPS (12 mg/kg) only (Fig. 4C). The mortality rate of LPS-administered mice groups, further treated with T15,21K-piscidin-1 or T15,21dK-piscidin-1 or polymyxin B were zero whilst only LPS-treated control mice group died within two days. Control mice group treated only with T15,21K-piscidin-1 (0.5 mg/kg) or T15,21dK-piscidin-1 (1.0 mg/kg) showed 100% survival ruling out any toxic effect of these peptides (data not shown). Survival of the mice was monitored for 7 days and the number of living/total mice for each treatment group is shown in Fig. 4C.

Further, to understand the basis of survival of LPS-administered mice in presence of these piscidin-1-analogs, the level of pro-inflammatory cytokines was measured in these mice. Significant inhibition of secretion of proinflammatory cytokines, TNF- α and IL-6 in serum of LPS-administered BALB/c mice was observed when they were treated with single dose of T15,21K-piscidin-1 (0.5 mg/kg) or T15,21dK-piscidin-1 (1 mg/kg) or polymyxin B (1 mg/kg), employed as a positive control (Fig. 4D). Levels of these cytokines in untreated and LPS-treated mice serum were taken as minimum and maximum to calculate the percentage of inhibition by piscidin-1-analogs.

### Atomic-resolution structures of piscidin-1, T15,21K-piscidin-1 and T15,21dK-piscidin-1 in LPS micelles

**Figure 5:**
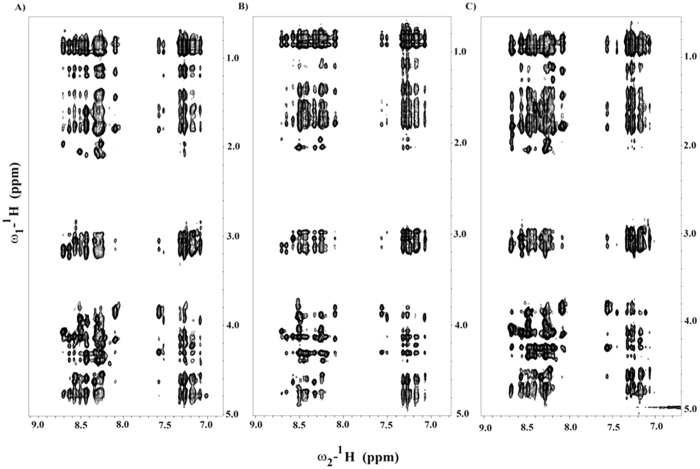
Selected sections of ^1^H-^1^H two-dimensional tr-NOESY spectra, obtained in the presence of LPS micelles, of piscidin-1 (panel A), T15,21K-piscidin-1 (panel B) and T15, 21dK-piscidin-1 (panel C) showing NOEs among low-field shifted amide and aromatic ring protons (along w2 dimension) with up-field shifted aliphatic protons (along w1 dimension).

**Figure 6:**
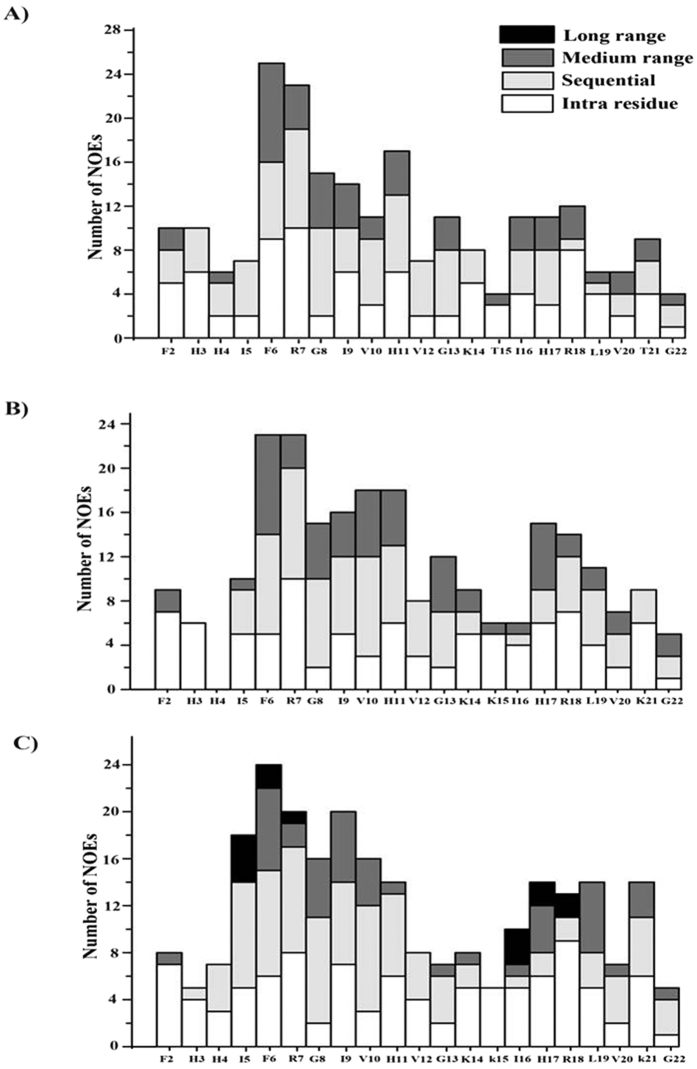
Bar diagrams summarizing type of NOEs observed for piscidin-1 (panel A), T15, 21K-piscidin-1 (panel B) and T15, 21dK-piscidin-1 (panel C) in LPS micelles.

**Figure 7:**
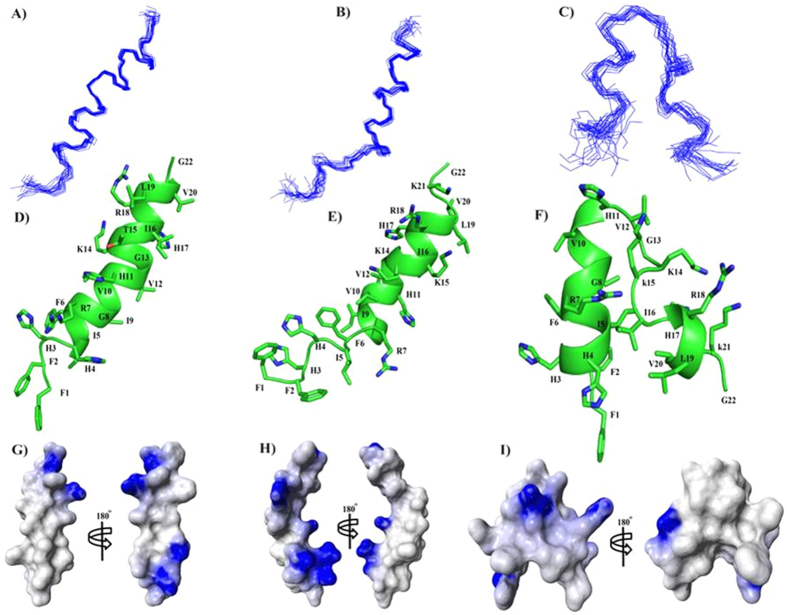
Superposition of twenty lowest energy structures of piscidin-1 (panel A), T15,21K-piscidin-1 (panel B) and T15,21dK-piscidin-1 (panel C) calculated using CYANA. Ribbon representations of a selected structure of piscidin-1 (panel D), T15,21K-piscidin-1 (panel E) and T15,21dK-piscidin-1 (panel F) showing backbone and sidechain orientation. Electrostatic surface diagrams of piscidin-1 (panel G), T15,21K-piscidin-1 (panel H) and T15,21dK-piscidin-1 (panel I) showing distribution of cationic charges (in blue) and non-polar residues (in white).

3-D structures of piscidin-1 and its analogs in complex with LPS were determined by two-dimensional ^1^H-^1^H tr-NOESY experiments^32^,^33^. Figure 5 shows selected sections of tr-NOESY spectra of piscidin-1 (panel A), T15,21K-piscidin-1 (panel B) and T15,21dK-piscidin-1 (panel C) showing NOE correlations among the low-field shifted amide and aromatic proton resonances (along w2 dimension) with the up-field shifted aliphatic proton resonances. Observations of a remarkably large number of NOEs in tr-NOESY spectra, demonstrate formation of folded structures of piscidin-1 and its two analogs upon binding to LPS micelles. Analyses of tr-NOESY spectra revealed presence of diagnostic medium range NOEs correlating resonances of backbone/backbone, backbone/sidechain and sidechain/sidechain for all three peptides (Fig. 6). Figure 7 shows superposition of backbone atoms (Cα, N and Cα) of twenty low energy structures of piscidin-1 (panel A), T15,21K-piscidin-1 (panel B) and T15,21dK-piscidin-1 (panel C). The structural statistics of the ensembles are listed in Table 3. As can be seen, piscidin-1 (panel D) and T15,21K-piscidin-1 (panel E) both assume well folded helical structures in LPS micelles. By contrast, T15,21dK-piscidin-1 analog has a distinctly different fold in LPS micelles (panel F). The helical structure of piscidin-1 spans from residue H4 to T21 with a kink at residue G13, whereas three N-terminal residues F1-F2-H3 of native piscidin-1 remain in largely extended conformation (panel D).

The electrostatic potential of piscidin-1 demarcates a large central hydrophobic surface with two smaller patches of cationic surfaces at the N- and C-termini (panel G). The helical structure of T15,21K-piscidin-1 which encompasses residues F6 to V20 with a kink at residue G13 appears to be shorter in length as compared to that of native piscidin-1. The side chains of a number of cationic residues including R7, H11, K14/K15, R18 and K21 are located along the one face of the helical structure of T15,21K-piscidin-1 (panel E). Whilst non-polar side chains of residues F6, I9/V10, V12, I16, and V20 can be seen reside at the other face of the helix (panel E). The electrostatic potential surface diagram of T15,21K-piscidin-1 delineates an extended positively charged region and a hydrophobic area at two different faces of the structure (panel H). The 3-D structure of T15,21dK-piscidin-1 is characterized by a short helix for N-terminal for residues H3-H11, followed by a turn or loop assumed by residues V12-I16 and a single turn of a 3_10_ helix consisted by residues H17-V20 (Panel F). The C-terminal loop structure packs against the N-terminal helix forming a hydrophobic core consisted by side chains of residues I5, I16, L19 and V20 (panel F). The 3-D topology of T15, 21dK of piscidin-1 discloses side chains of residues R7, K13, K14, D-Lys15 and R18 forms a cationic surface, whereas non-polar surface can be realized by the side chains of residues F2, I5, F6, F9 and V10 (Panel I).

## Discussion

Considering the significance of cell-selective, anti-microbial properties of AMPs for utilizing them as lead molecules for the development of novel natural antibiotics and immune-regulatory compounds^34^,^35^,^36^, in the present study several novel analogs of a naturally occurring AMP, piscidin-1 were designed. These analogs demonstrated antimicrobial as well as *in vitro* and *in vivo* anti-endotoxin properties with appreciably lower cytotoxicity. It is to be mentioned that recently we have designed and characterized several novel analogs of piscidin-1 by introducing single amino acid substitutions in a newly identified heptad repeat sequence in it ref. 14. However, in the present investigation we have demonstrated a different strategy to design its novel cell-selective analogs that exhibited improved *in vivo* anti-endotoxin activities. Though substitution of threonine residues with L- or D- lysine residues significantly reduced haemolytic and cytotoxic properties of piscidin-1 (Fig. 1A,B), its analogs displayed potent antibacterial (1–10 μM) activity against various Gram positive and Gram negative bacteria (Table 2) including several resistant strains of *S. aureus*. In the present investigation we have explored the amino acid substitutions in the interface of polar and non-polar faces of an AMP which is not well known in the literature for the design of its cell-selective analogs without compromising its antibacterial and anti-endotoxin properties. Piscidin-1 and its analogs induced depolarization of mammalian membrane mimetic, PC/Chol and bacterial membrane mimetic, PC/PG lipid vesicles (Fig. 1G,H) as well as damages of hRBC and bacterial membranes (Fig. 1C–F and Supplementary Figure S4) in presence of these peptides supported their relative antibacterial and cytotoxic activities respectively. The relative helical structures of antimicrobial peptides in bacterial and mammalian membrane mimetic environments have been often utilized to elucidate their relative antibacterial and cytotoxic properties^37^,^38^,^39^. As shown in Fig. 2 and Suppl. Table-S3 like cytotoxic properties, helical structures of piscidin-1 and its analogs in zwitterionic, PC/Chol lipid vesicles showed a significant variation (in the range of 50–9.5%). It is noteworthy that piscidin-1 and all its analogs adopted appreciable helical structures in negatively charged, PC/PG lipid vesicles and like antimicrobial properties, their helical structures varied within a shorter range (61–43%) (Suppl. Table-S3). The results altogether suggest that piscidin-1 and its analogs interact with the membrane of the mammalian or bacterial cell membrane to show their cytotoxic or antibacterial activities like other membrane-targeting AMPs^40^,^41^,^42^.

NMR studies revealed that the helical structure of T15,21K-piscidin-1 demonstrated an improved amphipathicity with an extended cationic surface compared to the parent peptide. In addition, in the helical structure the cationic side chains of residue R7, the guanidinium ^ε^N atom, and residue K15, ^ε^N atom, are separated by a distance of ~15 Å, which is optimally positioned to form ionic and/or hydrogen bonds with the bis-phosphate groups of the lipid A moiety of LPS^43^,^44^. Thus it could be inferred that placement of double L-lysine residues instead of two threonine residues (position 15 and 21) reinforced more amphipathic character in piscidin-1 that could led to its higher binding with LPS further resulting in its higher anti-endotoxin properties. Interestingly, introduction of two D-lysine residues caused significant conformational changes in piscidin-1 altering helical structure of piscidin-1 into helix-loop-helix structure. The different effects of these two stereoisomers (D and L) of lysine at the same position of piscidin-1 on the structure of piscidin-1 are very noteworthy. However, T15,21dK-piscidin-1 interacted with LPS appreciably as indicated by the LAL assay (Fig. 4A), ITC data (Supplementary Figure S5) and dissociation of LPS aggregates (Supplementary Figure S6). Notably, interactions of helix-loop-helix structure of T15,21dK-piscidin-1 with LPS could be maintained by the cationic side chains of residue R7 and residue D-Lys 15 showing a distance separation, between ^ε^N atoms, of ~13 Å, akin to the inter-phosphate distance of lipid A. The helix-loop-helix structures are also known for potent AMPs pardaxin, MSI-594 in LPS micelles that similarly bind to LPS/lipid A and showed antimicrobial and anti-endotoxin property^45^,^46^,^47^.

## Conclusions

Taken together, the results indicate an important role of the added two positively charged L or D-lysine residues in the interface of polar and non-polar faces of piscidin-1 for the generation of its two cell-selective analogs. These two novel analogs retained the antibacterial activity of their parent peptide and neutralized LPS-induced pro-inflammatory responses in THP-1 cells and demonstrated 100% survival of mice, treated with lethal dose of LPS in presence of their low doses. The NMR structures presented in this report are the first high resolution NMR structures of piscidin-1 and any of its analogs with LPS to our knowledge. The structural consequences of incorporation of D-amino acids in AMPs for their complex formation with bacterial lipids/membrane components remain largely unclear. The current study shows that inclusion of two D-lysine residues in the interface of piscidin-1, yielded folded structure of helix-loop-helix in LPS. Such information could be of significant importance for the design of anti-endotoxin peptides with a particular folding pattern. The newly designed two piscidin-1 analogs with additional double L- and D-lysine residues namely T15,21K-piscidin-1 and T15,21dK-piscidin-1 seem to possess significant attributes for considering them as potential lead molecules for the development of new antimicrobial/anti-sepsis agents.

## Materials and Methods

### Materials

Rink amide MBHA resin (loading capacity: 0.4–0.8 mmol/g), N-α-Fmoc and necessary side-chain protected amino acids were purchased from Novabiochem. Coupling reagents for peptide synthesis that include 1-hydroxybenzotriazole (HOBt), di-isopropylcarbodiimide (DIC), 1,1,3,3-tetramethyluronium tetrafluoroborate (TBTU) and N,N′-diisopropylethylamine (DIPEA) were purchased from Sigma, USA. Dichloromethane, N,N′-dimethylformamide (DMF) and piperidine were of standard grades and procured from reputed local companies. Acetonitrile (HPLC grade) was procured from Merck, India. Trifluoroacetic acid (TFA), N-[2-hydroxymethyl] piperazine-N′-[2-ethanesulfonic acid] (HEPES), sodium dodecyl sulfate (SDS), FITC-annexinV, valinomycin, dimethyl sulfoxide (DMSO) and cholesterol (Chol) were purchased from Sigma. Egg phosphatidylcholine (PC) and egg phosphatidylglycerol (PG) were obtained from Northern Lipids Inc., Canada whereas 3,3′-dipropylthiadicarbocyanine iodide (diS-C_3_–5), NBD-fluoride (4-fluoro-7-nitrobenz-2-oxa-1, 3-diazole) and tetramethylrhodaminesuccinimidyl ester were purchased from Invitrogen, India. *E.coli* 0111:B4 Lipopolysaccharide (L3012), FITC-LPS *E. coli* 0111:B4 (F3665) and Polymyxin B (P0972) from sigma. For Cell culture RPMI and Fetal Bovine Serum (EU-000-F) were purchased from sera laboratories west sussex UK. The GIBCO 100X Antibiotic–antimycotic (15240) was purchased from invitrogen corporations. Sterile polystyrene tissue culture flasks (690175), 96 well plates (655180) were procured from Greiner Bio-one, while 6 well plates (3506) were from Corning incorporated costar. Rests of the reagents of analytical grade were procured locally; buffers were prepared in milli Q water (USF-ELGA).

### Cytokine estimation kits

Human TNF-α (BD Biosciences cat. no. 555212) and Human IL-1β (BD Biosciences cat. no. 557953), Mouse TNF-α ELISA Set II (Cat: 558534) and Mouse IL-6 ELISA Set (Cat: 555240), used for ELISA experiments, were procured from BD OptEIA^TM^.

### Cell lines and animals

THP-1 and 3T3 cell lines were obtained from CSIR-Central Drug Research Institute, Lucknow cell line repository. The cell lines were maintained by usual protocol in an Innova CO2 incubator. Animals for experiments were provided by National Laboratory Animal Center, CSIR-CDRI (Lucknow). All animal procedures were carried out according to the protocols approved by the CSIR-central drug research institute Animal Ethics Committee No. IAEC/2010/79 and National Laboratory Animal Centre (Lucknow). Animals were properly anaesthetized before experiments and care was taken in all the animal experiments to minimize the sufferings to the animals. Our animal protocols were adhered to the guidelines of CPCSEA (Committee for the Purpose of Control and Supervision of Experiments on Animals), Govt. of India.

## Methods

### Peptide Synthesis, their Fluorescent labelling and Purification

It has been described in the Supplementary Methods section.

**“Computation of structural parameters”** has been described in Supplementary Methods section.

### Assay of haemolytic activity of the peptides

Haemolytic activity of the peptides against human red blood cells in PBS was performed by assaying the ability of the peptides to lyse the hRBCs^24^,^48^. For this purpose, fresh human red blood cells (hRBCs) were collected in the presence of an anti-coagulant from a healthy volunteer. After washing the blood three times with PBS haemolytic activity of the peptides were determined as reported previously^24^,^48^. Methodology of our experiment with human blood was in accordance with relevant guidelines and regulations of CSIR-central drug research institute ethics committee and was approved by it with approval No. CDRI/IEC/2014/A5. Further, informed consent was obtained from the healthy volunteer before collection of blood as per the guideline of our Institutional ethics committee.

### Antibacterial activity assay of the peptides

Antibacterial assays with all the peptides were done in 96-well microtiter plates against different Gram-positive, Gram-negative and resistant bacteria as described earlier^24^,^39^,^49^.

### Preparation of Small Unilamellar Vesicles (SUVs)

Small Unilamellar Vesicles were prepared by employing a bath type sonicator (Laboratory Supplies Company, New York) by a standard procedure with required amounts of either of the PC/cholesterol (8:1 w/w) or PC/PG (3:1 w/w) as described elsewhere^48^,^50^.

### Assay of peptide induced dissipation of diffusion potential

Peptide-induced permeabilization of lipid bilayer was measured by determining the ability to dissipate the diffusion potential across the lipid vesicles composed of either zwitterionic, PC/Chol (8:1, w/w) or negatively charged, PC/PG (3:1 w/w) by employing a potential sensitive probe diS-C_3_-5 as described in previous studies^26^,^51^.

### Circular dichroism (CD) studies

The circular dichroism (CD) spectra of peptide were recorded on Jasco J-815 spectropolarimeter in phosphate buffered saline (PBS, pH 7.4), zwitterionic PC/Chol (8:1, w/w), negatively charged PC/PG (3:1 w/w) lipid vesicles as described earlier^26^,^39^. Each spectrum was recorded as an average of three scans and cuvette of path length 2 mm was used for the experiments.

The fractional helicities (F_h_) of piscidin-1 and its analogs in lipid vesicles were calculated by the following formulae^52^,^53^


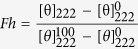


where [*θ*]_222_ was the experimentally observed mean residue ellipticity value at 222 nm. 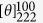 and 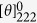 that correspond to 100 and 0% helix contents were considered to have mean residue ellipticity values of −32,000 and −2,000 respectively at 222 nm.

### Scanning Electron Microscopy (SEM)

The morphological changes induced by peptides on *E. coli* were studied using scanning electron microscopy as described earlier^54^. Micrographs were taken at magnifications of 12000× and 24000×. About 200 cells from two stubs for each sample were analyzed.

### Endotoxin neutralization assay (LAL assay)

The ability of peptides to bind LPS was assessed using a quantitative chomogenic limulus amoebocyte lysate (LAL) with QCL-1000 (LONZA 50-647U) kit as reported earlier^30^,^32^,^55^,^56^.

### Cell viability assay

Viability of the cells was determined to examine cytotoxicity of the peptides against murine 3T3 cells by a standard MTT assay as described earlier^39^,^42^,^57^.

### Measurement of cytokine expression levels in supernatant

It has been described in Supplementary Methods section.

***“In vivo*** **studies, treatment of Mice”** has been described in Supplementary Methods section.

### NMR and 3-D structure calculation

All NMR spectra were acquired, at 298 K, on a Bruker AVANCE II 600 MHz spectrometer, equipped with a cryo-probe and pulse field gradients. NMR data were processed and analysed by Topspin (Bruker) and SPARKY, respectively. A series of one dimensional proton NMR spectra of each peptide (0.75 mM in aqueous solution, PH 4.7) were recorded as a function of concentration of *E. coli* 0111:B4 LPS (MW 10 KD) ranging from 23 μM to 75 μM. Two-dimensional ^1^H-^1^H TOCSY (total correlation spectroscopy) and ^1^H-^1^H NOESY (nuclear Overhauser effect spectroscopy) spectra of all peptides were acquired in aqueous solutions (free peptide) with mixing times of 80 ms and 150 ms, respectively. Two dimensional ^1^H-^1^H NOESY or tr-NOESY spectra of piscidin-1 and its analogs were obtained in a mixture of peptide (0.75 mM) and LPS (50 μM) at mixing times of 100 and 150 ms. 3-D structures of LPS bound peptides were calculated using the CYANA 2.1. Based on the cross-peak intensities in the tr-NOESY spectra, upper bound distance limits were fixed to 2.5, 3.5, and 5.0 Å, corresponding to strong, medium, and weak intensities, respectively. During structure calculations the ϕ dihedral angles were constrained between −30 and −160° to maintain a good stereochemistry of the structures. Out of the 100 structures generated, the 20 lowest energy structures were used for further analysis.

**“Isothermal Titration Microcalorimetry experiment”** has been described in the Supplementary Methods section.

### Effect of peptide treatments on aggregated form of FITC-LPS

It has been described in the Supplementary Methods section.

### Detection of peptide-induced membrane damage of hRBCs and bacterial cells

It has been described in the Supplementary Methods section.

### Statistical Analysis

For statistical evaluation data were analysed using Prism5 (Graph Pad) software. For survival analysis Log Rank (Mantel- Cox) was used to determine significances (***p < 0.0001)^58^,^59^.

## Additional Information

**How to cite this article:** Kumar, A. *et al*. Piscidin-1-analogs with double L- and D-lysine residues exhibited different conformations in lipopolysaccharide but comparable anti-endotoxin activities. *Sci. Rep.* **7**, 39925; doi: 10.1038/srep39925 (2017).

**Publisher's note:** Springer Nature remains neutral with regard to jurisdictional claims in published maps and institutional affiliations.

## Supplementary Material

Supplementary Information

## Figures and Tables

**Table 1:** Sequence and molecular weight of piscidin-1 and its designed analogs.

Peptide	Sequence	Calculated mass	Observed mass	HPLC retention time
Piscidin-1	X-FFHHIFRGIVHVGKTIHRLVTG–amide	2572.38	2571.40	14.98
T15,21K Piscidin-1	X-FFHHIFRGIVHVGKKIHRLVKG–amide	2626.52	2625.30	13.40
T15,21dKpiscidin-1	X-FFHHIFRGIVHVGKkIHRLVkG–amide	2626.52	2625.30	12.38
T_15_K Piscidin-1	X-FFHHIFRGIVHVGKKIHRLVTG–amide	2599.45	2598.30	14.10
T_15_dK Piscidin-1	X-FFHHIFRGIVHVGKkIHRLVTG–amide	2599.45	2598.30	12.78

*Substituted amino acids are shown as underlined.

**k = D-Lysine; X = H or NBD.

**Table 2:** Antibacterial activity of piscidin-1 and its designed novel analogs.

Peptide	Minimum inhibitory conc. (MIC) in μM against bacteria										
	1	2	3	4	5	6	7	8	9	10	11
Piscidin-1	1.25 ± 0.3	2.5 ± 0.4	5.0 ± 0.7	2.5 ± 0.4	2.5 ± 0.4	2.5 ± 0.4	2.5 ± 0.4	6.0 ± 0.7	8.0 ± 1.0	3.8 ± 0.5	3.0 ± 0.3
T15,21K Piscidin-1	1.25 ± 0.3	2.5 ± 0.4	2.5 ± 0.4	2.5 ± 0.4	1.25 ± 0.3	2.5 ± 0.4	1.25 ± 0.3	5.0 ± 0.7	5.0 ± 0.7	3.0 ± 0.4	2.0 ± 0.3
T15,21dK Piscidin-1	5.0 ± 0.7	5.0 ± 0.7	10.0 ± 1.4	5.0 ± 0.7	5.0 ± 0.7	10.0 ± 1.4	5.0 ± 0.7	9.0 ± 1.3	9.0 ± 1.3	5.0 ± 0.7	5.0 ± 0.7
T15K Piscidin-1	1.25 ± 0.3	2.5 ± 0.4	2.5 ± 0.4	1.25 ± 0.3	1.25 ± 0.3	2.5 ± 0.4	2.5 ± 0.4	7.0 ± 1.0	8.5 ± 1.2	3.2 ± 0.4	3.5 ± 0.4
T15dK Piscidin-1	2.5 ± 0.4	2.5 ± 0.4	10.0 ± 1.4	2.5 ± 0.4	2.5 ± 0.4	5.0 ± 0.7	2.5 ± 0.4	7.5 ± 1.0	9.0 ± 1.3	3.6 ± 0.5	4.0 ± 0.6
Gentamycin	0.8	1.6	>100	>100	>50	>100	3.2	1.46	0.048	3.26	3.14
Norfloxacin	1.22	2.44	>150	2.44	39.0	>150	>150	2.19	0.39	0.125	0.125

MIC values are given as the means of three independent experiments each performed in duplicate ± the standard deviation.

*1. Staphylococcus aureus* (ATCC 25923), 2. *Staphylococcus aureus* (ATCC 29213), 3. *Staphylococcus aureus* (ATCC 700699 MRSA), 4. *Staphylococcus aureus* (ATCC 33592 *Gentamycin Resistant*), 5. *Staphylococcus aureus* (ATCC BAA-44 MRSA), 6. *Staphylococcus aureus* (ATCC 700698 MRSA), 7. *Staphylococcus aureus* (ATCC 1708 MRSA), 8. *P. aeruginosa* (ATCC BAA-427), 9. *B. subtilis* (ATCC 6633), 10. *K. pneumoniae* (ATCC 27736), 11. *E. coli* (ATCC 25922).

**Table 3:** Structural statistics of twenty lowest energy structures of piscidin-1 and its analogs.

Distance constraints	WT	L-Lys	D-Lys
Intra residue (i = j)	89	94	103
Sequential (i-j = 1)	43	44	45
Medium range [1<|i-j|≤4]	26	29	22
Long range [i-j > 4]	0	0	7
**Total**	158	167	177
**Deviation from mean structure**			
All backbone atoms (Å)	0.41	0.78	1.24
All heavy atoms (Å)	1.12	1.74	2.04
Ramachandran plot* for the mean structure			
% residues in the most favored region	72.2	72.2	72.2
% residues in the additionally allowed region	27.8	22.2	22.2
% residues in the generously allowed region	0.0	5.6	0.0
% residues in the disallowed region	0.0	0.0	5.6
*Calculated using Procheck			
**WT**: pisidin-1			
**L-Lys**: T15,21K-piscidin-1			
**D-Lys**: T15,21dK-piscidin-1			
