# Supplementary material for: Piscidin-1-analogs with double L- and D-lysine residues exhibited different conformations in lipopolysaccharide but comparable anti-endotoxin activities
Source: Sci Rep. 2017 Jan 4;7:39925. doi: 10.1038/srep39925 (PMC5209718; doi:10.1038/srep39925)
Supplement: Supplementary Information [file srep39925-s1.pdf]

Piscidin-1-analogs with double L- and D-lysine residues exhibited different conformations in lipopolysaccharide but comparable anti-endotoxin activities

Amit Kumar[1], Mukesh Mahajan[2], Bhanupriya Awasthi[3], Anshika Tandon[1], Munesh Kumar Harioudh[1], Sonal Shree[1], Pratiksha Singh[4], Praveen Kumar Shukla[4], Ravishankar Ramachandran[1], Kalyan Mitra[3], Surajit Bhattacharjya [2*] and Jimut Kanti Ghosh[1*].

[1]Molecular and Structural Biology Division, CSIR-Central Drug Research Institute,

Sector 10, Jankipuram Extension, Sitapur Road, Lucknow–226 031, India

[2]School of Biological Sciences, Nanyang Technological University, 60 Nanyang Drive, Singapore 637551, Singapore

[3]Electron Microscopy Unit, CSIR-Central Drug Research Institute,

Sector 10, Jankipuram Extension, Sitapur Road, Lucknow–226 031, India

[4]Microbiology Division, CSIR-Central Drug Research Institute,

Sector 10, Jankipuram Extension, Sitapur Road, Lucknow–226 031, India

*To whom correspondence should be addressed: Jimut Kanti Ghosh, Molecular and Structural Biology Division, CSIR-Central Drug Research Institute, Sector 10, Jankipuram Extension, Sitapur Road, Lucknow–226 031, India, Tel: 091-522-2771940 (Ext.-4451); Fax: 091-522-2771941, E-mail: jk_ghosh@cdri.res.in and Surajit Bhattacharjya, [2]School of Biological Sciences, Nanyang Technological University, 60 Nanyang Drive, Singapore 637551, Singapore, E-mail: surajit@ntu.edu.sg

Supplementary information

SupplementaryTable S1: Physicochemical properties of piscidin-1 and its analogs

Peptides	μH (Hydrophobic moment)	<H> (Hydrophobicity)	GRAVY
Piscidin-1	0.557	0.644	0.455
T15,21K-piscidin-1	0.612	0.530	0.164
T15K-piscidin-1	0.574	0.587	0.309

Mean hydrophobicity and hydrophobic moment were calculatedusing Heliquest software and net hydrophobic mean character was calculated by the GRAVY. D-lysine containing piscidin-1 analogs were not included in this Table since the concerned software is applicable for homochiral molecules only.

SupplementaryTable S2: Therapeutic index of piscidin-1 and its designed novel analogs

Peptide	HC_{50} (μM) hRBCs	MIC (μM) *E. coli*	Therapeutic Index (HC_{50}/MIC)
**Piscidin-1**	**15**	**3.0**	**5.00**
**T15,21K-piscidin-1**	**200**	**2.0**	**100**
**T15,21dK-piscidin-1**	**500**	**5.0**	**100**
**T15K – piscidin-1**	**46**	**3.5**	**13.14**
**T15dK – piscidin-1**	**125**	**4.0**	**31.25**

HC_{50}= Peptide concentration leading to 50% lysis of human RBC.

MIC = Minimum inhibitory concentration of peptide against *E. coli* (ATCC 25922).

Supplementary Table S3: Determined percentages of α-helical structures of piscidin-1 and its analogs in different lipid vesicles

Peptide	PC/Chol		PC/PG	
	$[\theta]_{222}$	% α-Helix	$[\theta]_{222}$	% α-Helix
Piscidin-1	$-17.0897x10^{3}$	50.29	$-20.3062x10^{3}$	61.02
T15,21K Piscidin-1	$-10.2015x10^{3}$	27.33	$-19.8774x10^{3}$	59.59
T15,21dK piscidin-1	$-4.8541x10^{3}$	9.51	$-14.8987x10^{3}$	42.99
T15K Piscidin-1	$-15.9788x10^{3}$	46.59	$-19.9674x10^{3}$	59.89
T15dK Piscidin-1	$-10.4104x10^{3}$	28.03	$-15.6214x10^{3}$	45.40

Supplementary Figure S1

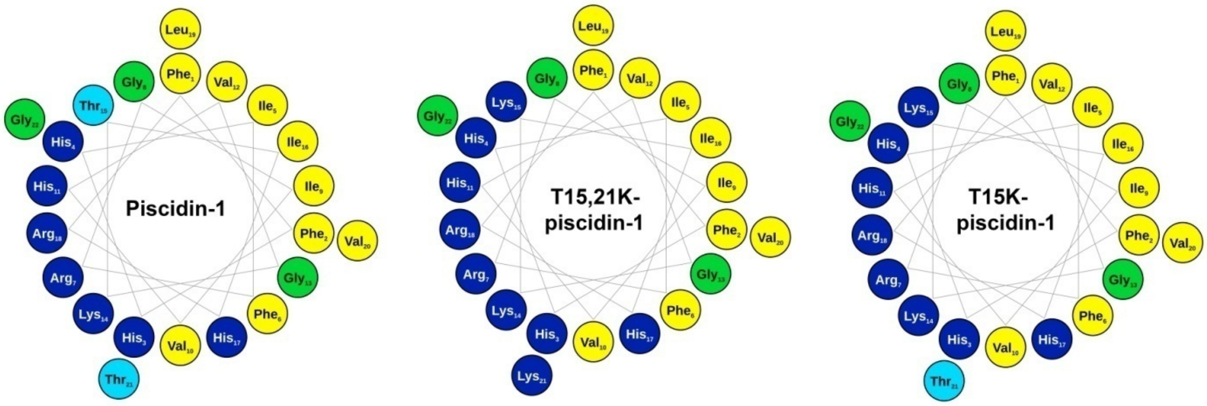

Helical wheel projections of naturally occurring piscidin-1 and its designed analogs. The helical wheels were generated using ProteinOrigami tool. Amino acids in yellow circle: non-polar in nature;green circle: neither polar nor non-polar; blue circle: Charged and polar in nature.

Supplementary FigureS2

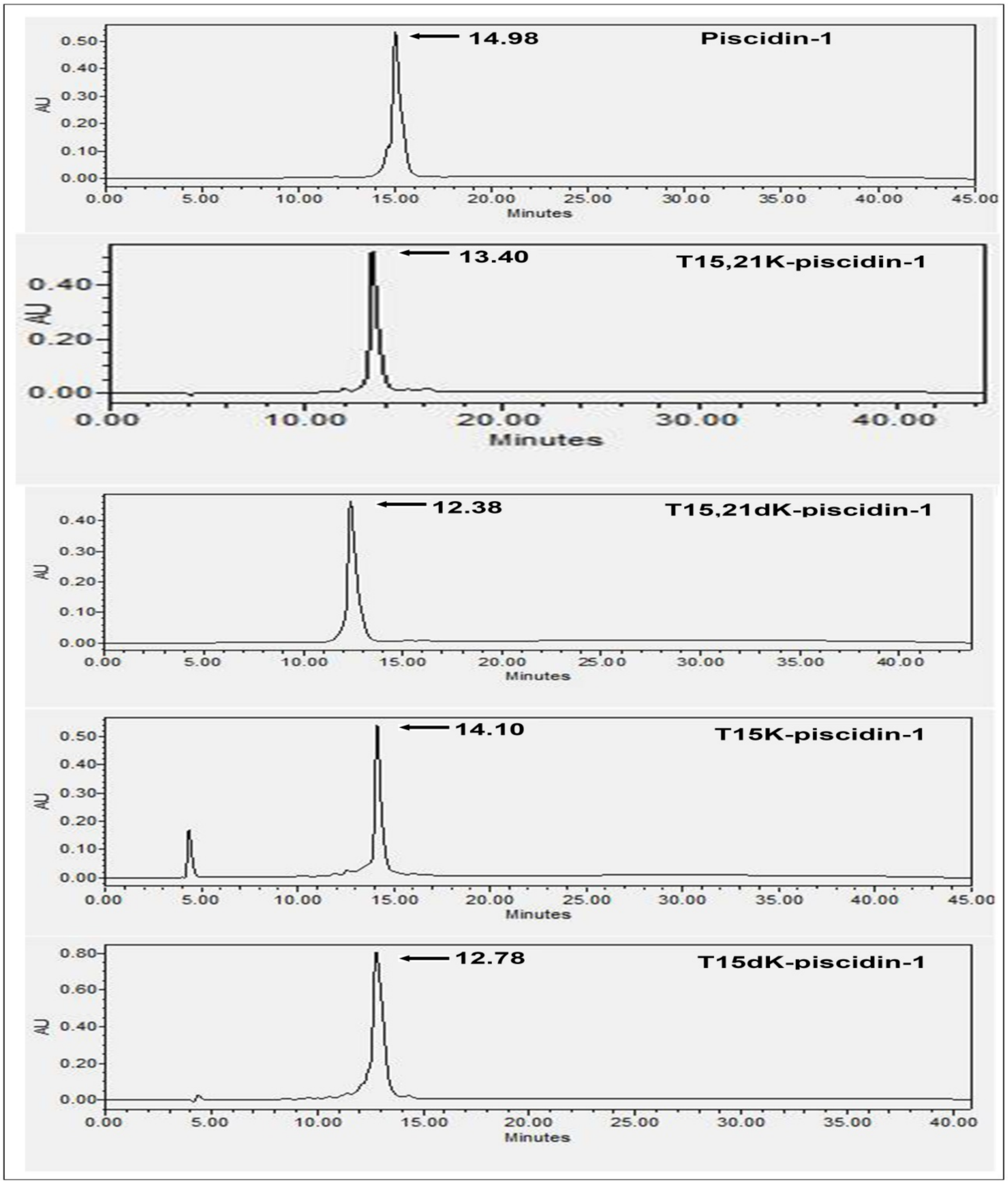

HPLC profiles for purification of piscidin-1 and its analogs. Purifications by reverse phase HPLC were accomplished by a gradient of water and acetonitrile (95:05) in 40 minutes with a semi-preparative C18 Waters DeltaPak HPLC column (particle size, 15µm; pore size, $300A^0$; ID, 7.8 mm; length 300 mm). Arrow indicates the retention time of each of the peptide.

Supplementary FigureS3

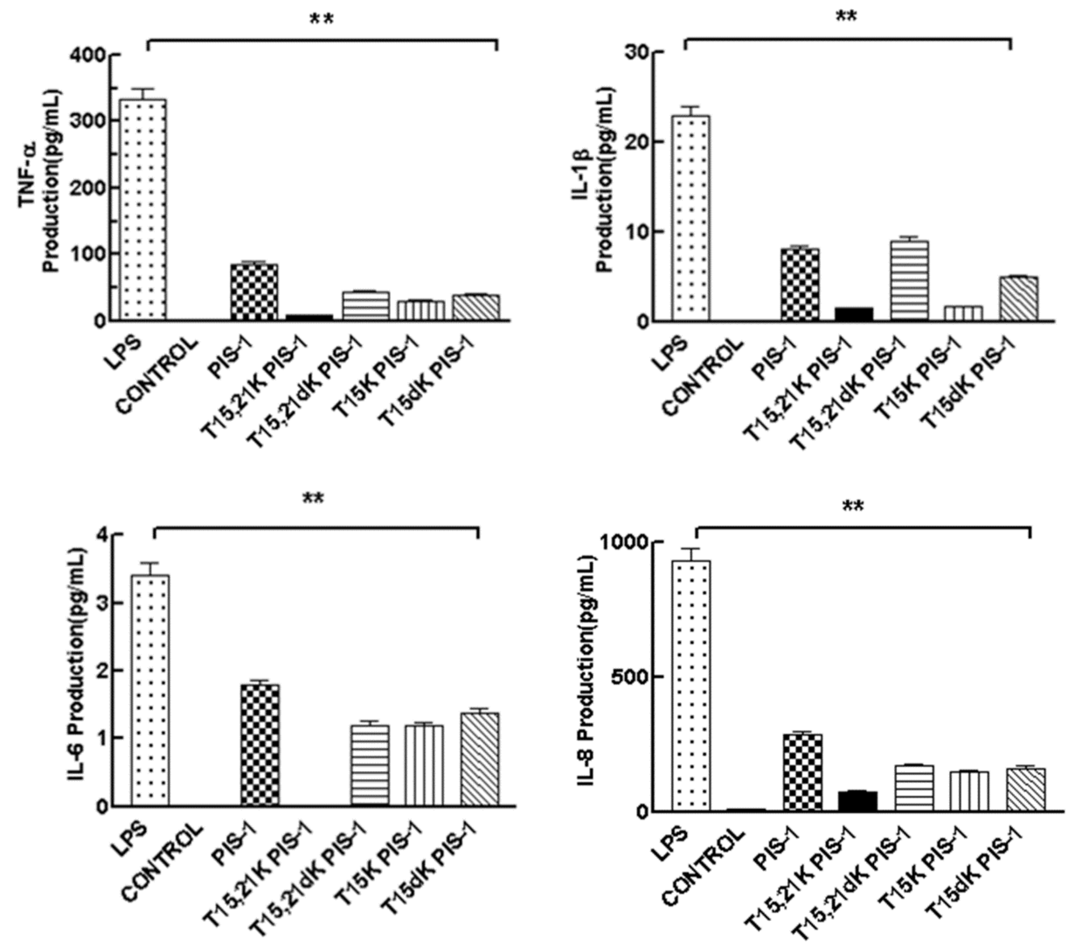

Effect of piscidin-1 and its analogs onto the secretion of proinflammatory cytokines in LPS-stimulated THP-1 cells. THP-1 cells were incubated in absence or presence of LPS and piscidin-1 and its designed analogs for 6 hours. The concentrations of secreted TNF-α, IL-1β, IL-6 and IL-8 in cell culture supernatant were estimated by BD bioscience cytometry Bead Array.Statistical analysis was carried out using one-way analysis of variance using Tukey's Test. P-values of ≤ 0.01, ≤ 0.001 and ≤ 0.0001 between different groups were considered as significant, highly significant and very highly significant and marked with *, ** and * respectively.**

Supplementary FigureS4

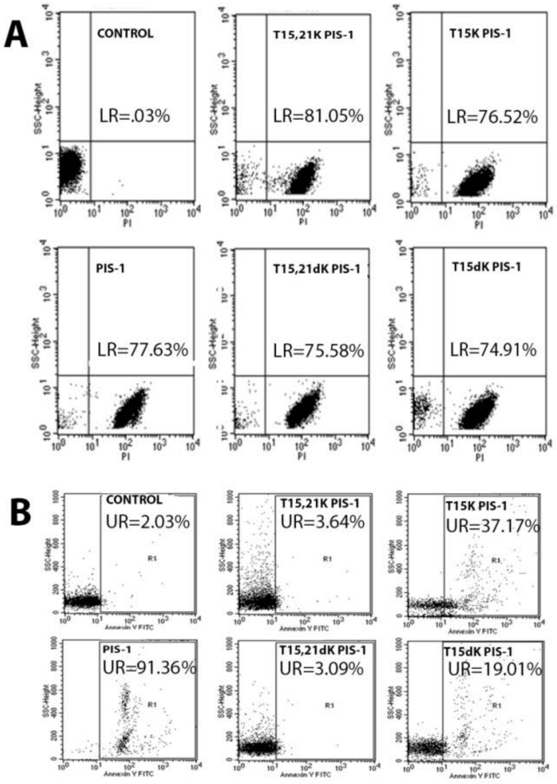

(A) Peptide-induced membrane damage of *E. coli* ATCC25922 as detected by PI staining following the treatment of different peptides.Propidium iodide (PI) binds to nucleic acids, which is possible only after the damage of bacterial membrane. Piscidin-1 and its analogs induced extensive PI staining of *E. coli* indicating bacterial membrane damaging properties of these peptides. Lower left quadrant of each panel depicts unstained cells whereas the lower right quadrant depicts the stained cells. Concentrations of the peptides were ~5.0 µM. (B) Peptides induced damage of hRBCs membrane as detected by FITC-annexin V staining. Peptide-induced damage of hRBCs leads to exposure of phosphatidylserine to the annexinV-FITC, resulting in the staining of hRBCs which is directly proportional to membrane damaging property of the peptides. Left quadrant of each panel depicts unstained cells, whereas the right quadrant depicts the stained cells. Concentrations of the peptides were 25.0 µM. 10000 events were recorded for each sample and control panels show the negligible PI and FITC-annexin V staining of the bacteria and hRBCs respectively in the absence of any peptide.

Supplementary FigureS5

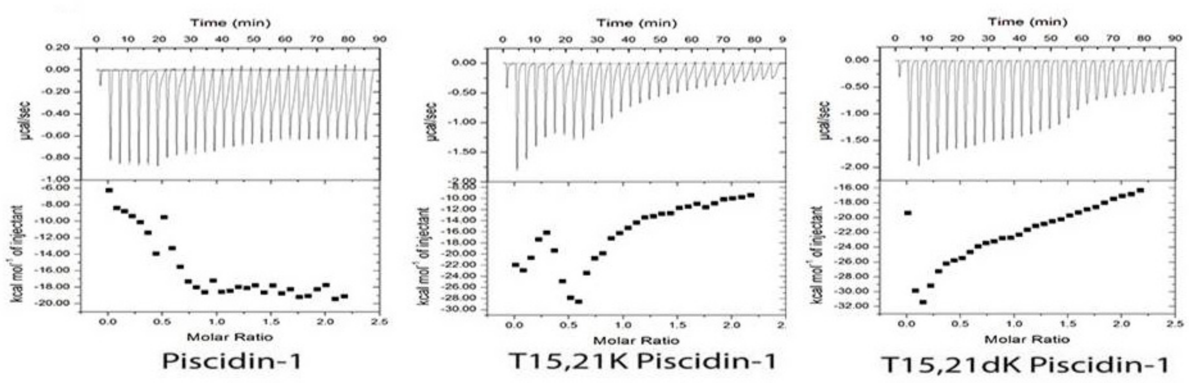

Isothermal titration calorimetry (ITC) profiles for studying the interaction of piscidin-1, T15,21K-piscidin-1 and T15,21dK-piscidin-1 with LPS.Concentrations of LPS and peptide were 25 and 250 µM respectively and experiments were conducted at around physiological temperature (38°C) on which LPS is supposed to be in the liquid crystalline phase.When LPS O111:B4 was titrated with piscidin-1 and their analogs, typical exothermic isotherms were obtained for all the peptides that were evident from the downward titration peaks and negative values of the integrated heats. However, the binding curves appeared to be complex, probably associated with multiple binding steps and therefore thermodynamic parameters were not determined from quantitative analyses.[1] Exothermic nature of the binding curves is an indication that interaction of piscidin-1 and its analogs with LPS was enthalpy driven which further suggested a prominent role of polar interactions involving ionic and hydrogen bond formation between these peptides and LPS.

Supplementary FigureS6

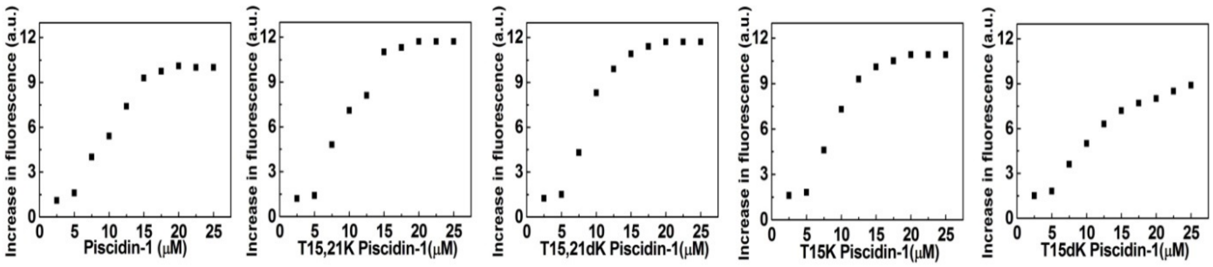

Dissociation of FITC-LPS aggregates in the presence of increasing concentrations of piscidin-1 and its analogs.Piscidin-1 dissociated FITC-LPS aggregates as indicated by the dequenching of FITC-LPS fluorescence with increase in peptide concentration. However, T15,21K-piscidin-1 and T15,21dK-piscidin-1 were found to some extent more persuasive than piscidin-1 in inducing disaggregation in FITC-LPS aggregates.

Supplementary Methods:

Peptide Synthesis, their Fluorescent labelling and Purification

Peptide synthesis was done manually utilizing solid phase method on rink amide MBHA resin using Fmoc chemistry as described previously[2-8]. Labelling of the peptides at their N-terminus by NBD was achieved by a usual procedure as described earlier [9]. Cleavage of labelled and unlabelled peptides from resins and their purification by reverse phase HPLC was accomplished as reported previously[9,10].

Computation of structural parameters

By using Heliquest software, basic hydrophobicity parameters such as mean hydrophobicity and hydrophobic moment were calculated. The peptide's net hydrophobic mean character was calculated by the GRAVY scale[11].

Measurement of cytokine expression levels in supernatant

Enzyme linked immunosorbent assays were carried out to estimate the secreted TNF-α and IL-1β in LPS treated cells in presence of the peptides after 4-6 hrs incubation. Levels of these cytokines in culture supernatant of untreated and LPS-treated cells were taken as minimum and maximum for working out the percentage inhibition by peptides[12]. Concentrations of TNF-α and IL-1β in the samples were assessed using human enzyme linked immunosorbent assay kits for TNF-α (BD Biosciences cat.no.555212) and IL-1β (BD Biosciences cat. no. 557953) according to manufacturers' protocol. The experiments were repeated thrice, and the average values of the determined cytokine concentrations were included in the results. Similar ELISA experiments were performed to estimate the secreted TNF-α and IL-6 with BALB/c mice blood collected by orbital sinus 4 hr post LPS injection by using mouse enzyme linked immunosorbent assay kits for TNF-α (BD Biosciences cat.no.558534) and IL-6 (BD Biosciences cat. no. 555240).

Alternatively, four cytokines (TNF-α, IL-1β, IL-6 and IL-8) were measured simultaneously using Human Inflammatory Cytokine Kit from BD Cytometric Bead Array (CBA, cat.no. 551811) as per the manufacturer's instructions. The multiplex bead immunoassays were performed using BD Biosciences FACS Calibur flow cytometer system. BD CBA Human Inflammatory Cytokines Kit data analyzed using FCAP Array software.

***In vivo* studies, treatment of Mice**

Female BALB/c mice (National laboratory animal centre, central drug research institute, Lucknow) were given a standard laboratory diet and water *ad libitum* and housed under controlled environmental conditions. The mice were approximately 25 g each at the start of experiments. All the mice were divided into experimental groups for the administration of LPS and peptides. Mice were treated with 12 mg/kg *E. coli* 0111:B4 LPS in presence and in absence of peptide in different experimental groups. The mice treated with saline only, in absence of LPS were considered as experimental controls. To study the efficacy of a peptide in neutralizing LPS-induced pro-inflammatory responses in mice ~5 min post LPS treatment, the respective peptide was administered i.p. at a site other than the one, used for LPS injection. [13-15]. Survival of mice was monitored for 7 days. Survival of mice was also monitored following the treatment of only peptides for investigating their toxic effects.

Isothermal Titration Microcalorimetry:

ITC experiments were carried out by employing a VP-ITC instrument (GE). Injections of 10 μl of peptide solution were added from a computer-controlled micro syringe at 3 min intervals into the LPS suspension (cell volume 1.43 ml) with stirring at 350 rpm. Titration experiments were performed at pH 7 using 20 mM HEPES. LPS and respective peptide concentrations were 25 and 250 μM respectively. Experimental data were tried to be fitted with various theoretical titration curves/models by utilizing software supplied by Microcal. The quantity $c= K_aM_t$, where Mt, the initial macromolecule concentration, is important in titration Microcalorimetry[16]. For titration experiments c values were taken in the range of $1<c<200$. The instrument was routinely calibrated with the help of calibration kit supplied by the manufacturer.

Effect of peptide treatments on aggregated form of FITC-LPS:

The assay was carried out as described previously [17-19]. FITC-LPS (0.5 μg/mL) was treated with increasing concentrations of a peptide. The changes in the emission of FITC (515 nm) in 10 mM sodium phosphate buffer, pH 6.9, were monitored by a fluorescence spectrometer (Perkin Elmer LS55) with excitation wavelength set at 488 nm and excitation and emission slits of 8 and 6 nm respectively. The emissions of both sodium phosphate buffer and a peptide alone due to scattering

of light were taken for background subtractions. Dissociation of LPS-aggregates results in an increase in the fluorescence of FITC fluorophore, attached to it [18,20].

Detection of peptide-induced membrane damage of hRBCs and bacterial cells:

Peptide-induced damage of membrane organization of hRBCs was determined by staining the cells (~3.0×10^7 cells /ml) with FITC-annexinV[21,22] after the treatment with the peptides at room temperature for 10-15 min. Extent of staining of cells was measured by analyzing peptide-treated cells with respect to peptide untreated control cells, using Becton Dickinson FACS Calibur flow cytometer and Cell Quest Pro software.

To examine the peptide-induced damage to membrane integrity of *E. coli* ATCC 25922, the cells at mid-log phase were incubated with a peptide, under investigation, for 1 hour at 37°C with constant shaking followed by their washing for two times with PBS, and incubated further with propidium iodide at 4°C for 30 min. After removal of the unbound propidium iodide through washing with an excess of PBS and re-suspended in buffer, peptide-induced damage of bacterial cells was analyzed by the flow cytometer as mentioned above.

1 Shrivastava, T., Dey, A. & Ramachandran, R. Ligand-induced structural transitions, mutational analysis, and 'open' quaternary structure of the M. tuberculosis feast/famine regulatory protein (Rv3291c). *J Mol Biol***392**, 1007-1019, doi:10.1016/j.jmb.2009.07.084S0022-2836(09)00931-0 (2009).

2 Asthana, N., Yadav, S. P. & Ghosh, J. K. Dissection of antibacterial and toxic activity of melittin: a leucine zipper motif plays a crucial role in determining its hemolytic activity but not antibacterial activity. *J BiolChem***279**, 55042-55050, doi:M408881200 10.1074/jbc.M408881200 (2004).

3 Ahmad, A. *et al.* Utilization of an amphipathic leucine zipper sequence to design antibacterial peptides with simultaneous modulation of toxic activity against human red blood cells. *J BiolChem***281**, 22029-22038, doi:M602378200 10.1074/jbc.M602378200 (2006).

4 Pandey, B. K. *et al.* Cell-selective lysis by novel analogues of melittin against human red blood cells and Escherichia coli. *Biochemistry***49**, 7920-7929, doi:10.1021/bi100729m (2010).

5 Tripathi, J. K. *et al.* Variants of self-assembling peptide, KLD-12 that show both rapid fracture healing and antimicrobial properties. *Biomaterials***56**, 92-103, doi:10.1016/j.biomaterials.2015.03.046S0142-9612(15)00324-5 (2015).

6 Fields, G. B. & Noble, R. L. Solid phase peptide synthesis utilizing 9-fluorenylmethoxycarbonyl amino acids. *Int J Pept Protein Res***35**, 161-214 (1990).

7 Wild, C., Oas, T., McDanal, C., Bolognesi, D. & Matthews, T. A synthetic peptide inhibitor of human immunodeficiency virus replication: correlation between solution structure and viral inhibition. *Proc Natl AcadSci U S A***89**, 10537-10541 (1992).

8 Bouchayer, E., Stassinopoulou, C. I., Tzougraki, C., Marion, D. &Gans, P. NMR and CD conformational studies of the C-terminal 16-peptides of Pseudomonas aeruginosa c551 and Hydrogenobacterthermophilus c552 cytochromes. *J Pept Res***57**, 39-47, doi:jpr792 (2001).

9 Azmi, S. *et al.* Characterization of antimicrobial, cytotoxic, and antiendotoxin properties of short peptides with different hydrophobic amino acids at "a" and "d" positions of a heptad repeat sequence. *J Med Chem***56**, 924-939, doi:10.1021/jm301407k (2013).
10 Tripathi, J. K., Kathuria, M., Kumar, A., Mitra, K. & Ghosh, J. K. An unprecedented alteration in mode of action of IsCT resulting its translocation into bacterial cytoplasm and inhibition of macromolecular syntheses. *Sci Rep***5**, 9127, doi:10.1038/srep09127srep09127 (2015).
11 Torrent, M., Andreu, D., Nogues, V. M. &Boix, E. Connecting peptide physicochemical and antimicrobial properties by a rational prediction model. *PLoS One***6**, e16968, doi:10.1371/journal.pone.0016968 (2011).
12 Mangoni, M. L. *et al.* Lipopolysaccharide, a key molecule involved in the synergism between temporins in inhibiting bacterial growth and in endotoxin neutralization. *J BiolChem***283**, 22907-22917, doi:M800495200 10.1074/jbc.M800495200 (2008).
13 Mitsui, S., Hidaka, C., Furihata, M., Osako, Y. & Yuri, K. A mental retardation gene, motopsin/prss12, modulates cell morphology by interaction with seizure-related gene 6. *BiochemBiophys Res Commun***436**, 638-644, doi:10.1016/j.bbrc.2013.04.112S0006-291X(13)00964-9 (2013).
14 Kalle, M. *et al.* A peptide of heparin cofactor II inhibits endotoxin-mediated shock and invasive Pseudomonas aeruginosa infection. *PLoS One***9**, e102577, doi:10.1371/journal.pone.0102577 PONE-D-14-05351 (2014).
15 Kumar, A. *et al.* Single amino acid substitutions at specific positions of the heptad repeat sequence of piscidin-1 yielded novel analogs that show low cytotoxicity as well as in vitro and in vivo anti-endotoxin activity. *Antimicrob Agents Chemother*, doi:AAC.02341-15 10.1128/AAC.02341-15 (2016).
16 Holdgate, G. Isothermal titration calorimetry and differential scanning calorimetry. *Methods Mol Biol***572**, 101-133, doi:10.1007/978-1-60761-244-5_7 (2009).
17 Bhunia, A., Mohanram, H. &Bhattacharjya, S. Lipopolysaccharide bound structures of the active fragments of fowlicidin-1, a cathelicidin family of antimicrobial and antiendotoxic peptide from chicken, determined by transferred nuclear Overhauser effect spectroscopy. *Biopolymers***92**, 9-22 (2009).
18 Srivastava, S. & Ghosh, J. K. Introduction of a lysine residue promotes aggregation of temporin L in lipopolysaccharides and augmentation of its antiendotoxin property. *Antimicrob Agents Chemother***57**, 2457-2466, doi:10.1128/AAC.00169-13AAC.00169-13 (2013).
19 Srivastava, R. M., Srivastava, S., Singh, M., Bajpai, V. K. & Ghosh, J. K. Consequences of alteration in leucine zipper sequence of melittin in its neutralization of lipopolysaccharide-induced proinflammatory response in macrophage cells and interaction with lipopolysaccharide. *J BiolChem***287**, 1980-1995, doi:10.1074/jbc.M111.302893M111.302893 (2012).
20 de Haas, C. J., van Leeuwen, H. J., Verhoef, J., van Kessel, K. P. & van Strijp, J. A. Analysis of lipopolysaccharide (LPS)-binding characteristics of serum components using gel filtration of FITC-labeled LPS. *J Immunol Methods***242**, 79-89 (2000).
21 Kuypers, F. A. *et al.* Detection of altered membrane phospholipid asymmetry in subpopulations of human red blood cells using fluorescently labeled annexin V. *Blood***87**, 1179-1187 (1996).
22 Yadav, S. P., Ahmad, A., Pandey, B. K., Verma, R. & Ghosh, J. K. Inhibition of lytic activity of Escherichia coli toxin hemolysin E against human red blood cells by a leucine zipper peptide and understanding the underlying mechanism. *Biochemistry***47**, 2134-2142, doi:10.1021/bi701187e (2008).
